# Correction to: PEMT: a patent enrichment tool for drug discovery

**DOI:** 10.1093/bioinformatics/btad758

**Published:** 2023-12-21

This is a correction to: Yojana Gadiya, Andrea Zaliani, Philip Gribbon, Martin Hofmann-Apitius, PEMT: a patent enrichment tool for drug discovery, *Bioinformatics*, Volume 39, Issue 1, January 2023, btac716, https://doi.org/10.1093/bioinformatics/btac716

In the originally published version of this manuscript, the information provided in the Funding section is not accurate.

Incorrect Funding Details:

This work was supported by the German Federal Ministry of Education and Research (BMBF) [01GM2003A] (project ‘TreatKCNQ’).

Correct Funding Details:

This work was supported by the German Federal Ministry of Education and Research (BMBF) [01GM2003A] (project ‘TreatKCNQ’) and EOSC-Life which has received funding from the European Union's Horizon 2020 programme under grant agreement number 824087.

This error has been corrected online.

