# Supplementary figures and images for: PEMT: a patent enrichment tool for drug discovery
Source: Bioinformatics. 2022 Nov 2;39(1):btac716. doi: 10.1093/bioinformatics/btac716 (PMC9805556; doi:10.1093/bioinformatics/btac716)

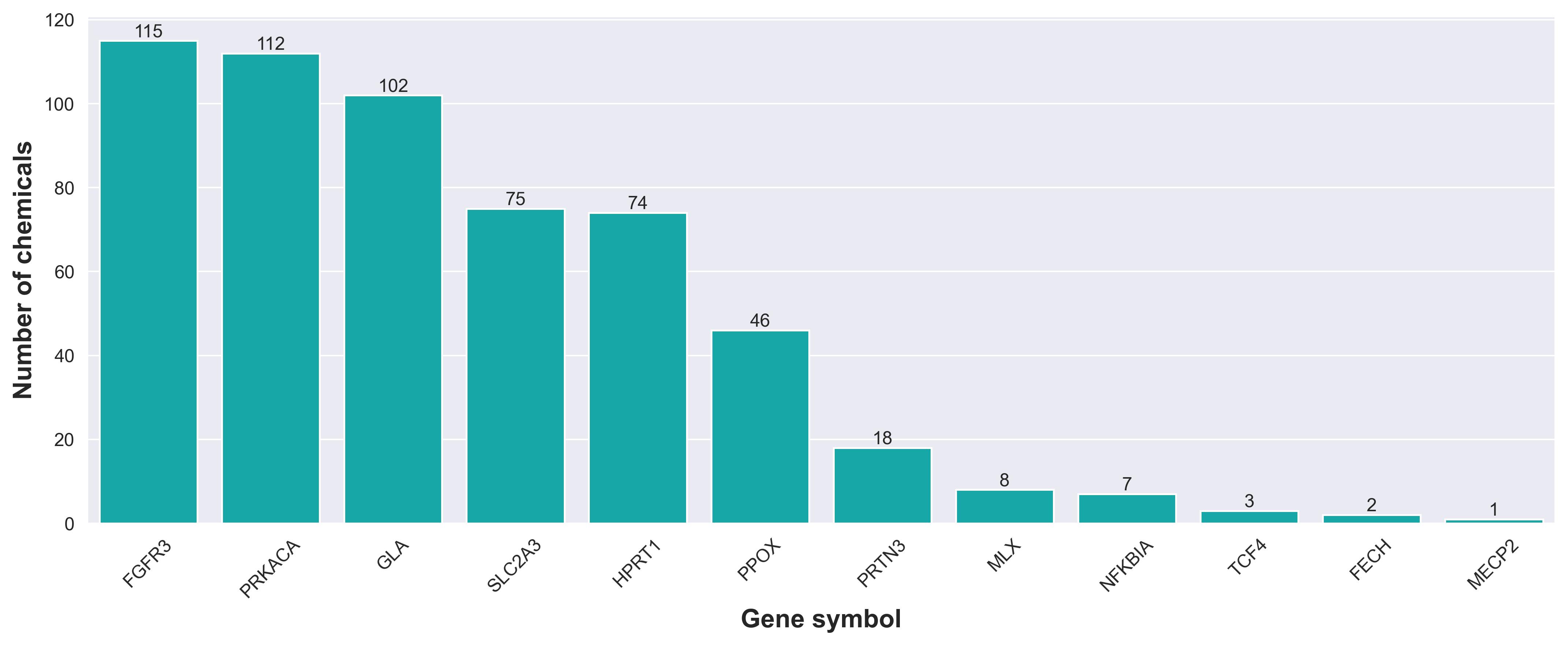

Supplement: btac716_Supplementary_Data [file btac716_supplementary_data.zip › btac716_Supplementary_Data/supplement_figure_1.jpg]

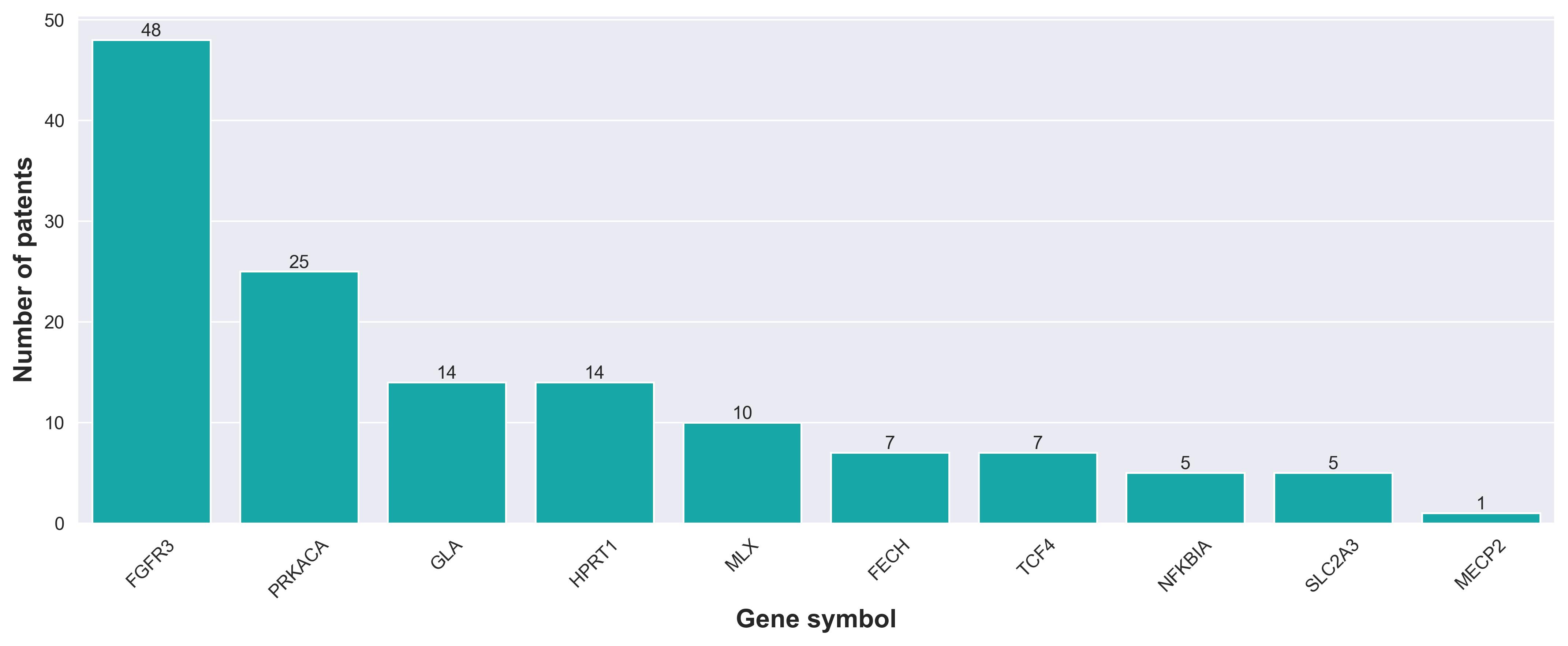

Supplement: btac716_Supplementary_Data [file btac716_supplementary_data.zip › btac716_Supplementary_Data/supplement_figure_2.jpg]

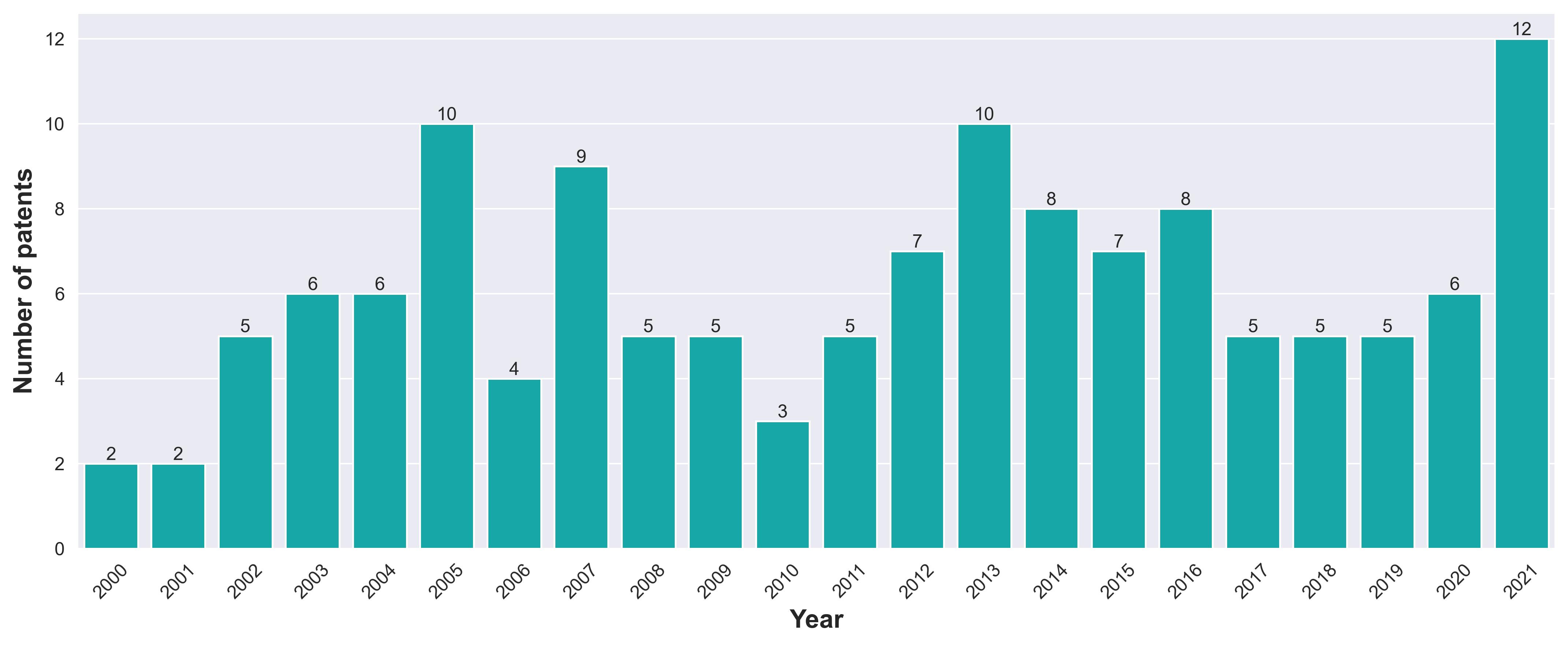

Supplement: btac716_Supplementary_Data [file btac716_supplementary_data.zip › btac716_Supplementary_Data/supplement_figure_3.jpg]

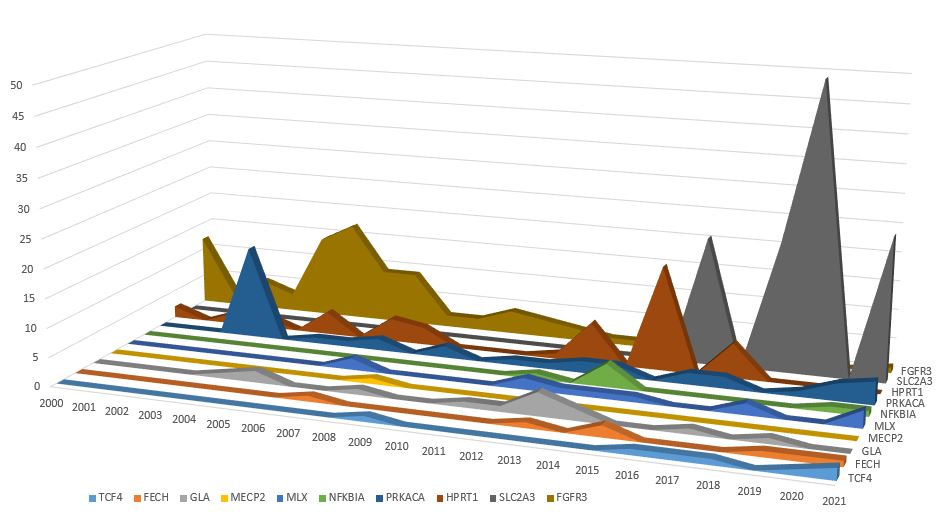

Supplement: btac716_Supplementary_Data [file btac716_supplementary_data.zip › btac716_Supplementary_Data/supplement_figure_4.tif]
